# Mechanical performance of structural lightweight concrete with metallurgical coal aggregates

**DOI:** 10.1038/s41598-026-37929-6

**Published:** 2026-02-20

**Authors:** Toka Waleed, Mohammed Rady, Ibrahim Mohsen Mashhour, Mohammed El-Attar

**Affiliations:** 1 Construction and Building Engineering Department, College of Engineering and Technology, Arab Academy for Science, Technology and Maritime Transport (AASTMT), B 2401 Smart Village, Giza, 12577 Egypt; 2 Department of Structure Engineering, Faculty of Engineering, Cairo University, Giza, Egypt

**Keywords:** Lightweight aggregates, X-ray diffraction, Elevated temperature, Geochemical analysis, Compressive strength, Engineering, Materials science

## Abstract

Many studies have investigated industrial by-products as alternatives to natural aggregates; however, little attention has been given to metallurgical coal (MC). This study evaluates MC as a replacement for coarse aggregate to develop structural lightweight concrete (SLWC), using five mixtures with MC contents of 0%, 25%, 50%, 75%, and 100% by weight. To this end, an experimental program on testing the microstructural and chemical analysis of MC and the mechanical properties of fresh and hardened concrete was conducted. The results showed that increasing MC reduced mechanical performance; compressive (flexural) strength decreased from 37.6 (5.17) MPa at 0% MC to 20.7 (2.75) MPa at 100% MC. Furthermore, increasing the MC content reduced the elastic modulus per four international codes. On the other hand, the results showed that MC significantly decreased density, achieving up to a 24.3% reduction relative to normal-weight concrete and satisfying lightweight classification. Moreover, concrete mixtures with 25% and 50% MC maintained adequate structural performance when subjected to elevated temperatures. From an economic perspective, 75% MC lightweight concrete was more cost-effective and required 12% less reinforcing steel than normal-weight concrete. These findings indicate that MC can improve natural resource sustainability and offer a cost-effective route for sustainable construction.

## Introduction

In the past several decades, structural lightweight concrete (SLWC) has played a crucial role in construction. The three most popular structures were built during the early Roman Empire, including the Port of Cosa, the Pantheon Dome, and the Coliseum^1^. Nowadays, SLWC is widely used in major construction projects, especially when it shows a total reduction in the dead load of the structural elements of the buildings. In comparison with normal-weight concrete (NWC), SLWC is a cost-effective concrete, due to its lighter weight, and reduction in transportation costs as it requires less equipment and manpower, and helps in saving energy^2^. Concrete’s performance and quality depend on its constituent ingredients, including aggregate. Aggregate is a crucial component of concrete, a filler of 60% to 80% of the concrete^3^. The existing literature indicates that using waste as a lightweight aggregate enhances the properties of concrete and provides a sustainable way to reduce waste globally^4^.

Building on this foundation, sustainable concrete construction increasingly relies on incorporating by-products as partial replacements for Portland cement and natural aggregates. For instance, a recent study^5^ utilized waste tea ash and sugar beet waste ash^6^, while another study used peanut and sunflower shell ash to perform sustainable lightweight concrete using agricultural by-products. Recent studies have highlighted the reuse of various industrial and solid waste by-products in performing structural lightweight concrete. For example, Sathe and Rathod^7^ investigated structural lightweight mortars incorporating waste PET aggregates. Kalane et al.^8^ reported that steel slag was used as coarse aggregate in SLWC. Gaidhankar et al.^9^ examined marble waste aggregate in concrete with metakaolin. Recent advances in concrete technology consider the usage of coal in the manufacturing of concrete to enhance sustainable development goals. The most popular type of coal is thermal coal, as its name signifies, used to generate heat energy. However, it is also utilized in the production of cement and other industries. The other type is metallurgical coal (MC), which is a fossilized material found only on Earth that can be used to produce good-quality coke, which is an essential fuel in the process of blast furnaces used for steel-making^10^. MC is usually combined with iron ore to make steel, it consists mainly of high carbon content, porosity, and the physical transformation during the coking process, which makes it technically suitable for lightweight aggregate production.

**Fig. 1:**
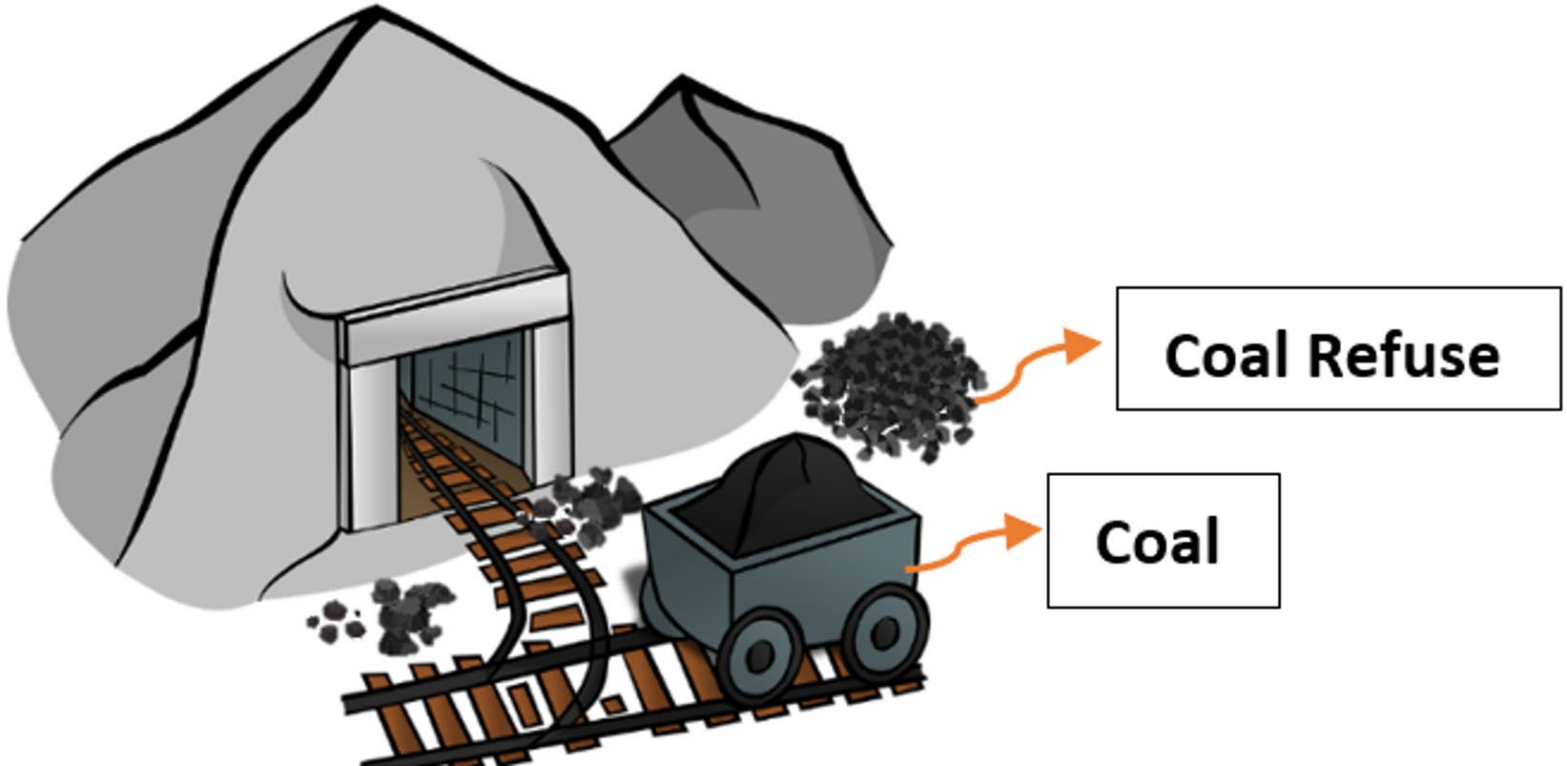
Coal mine and coal mine refuse.

It is widely considered that the environment and public health are adversely affected by waste materials, such as coal mine waste rock known as “coal refuse,” as shown in Fig. 1. Thus, sustainable development must use leftover rock from coal mining to replace aggregate in the production of concrete^11^.

Recent studies have explored strategies to mitigate environmental damage from untreated coal waste (UCW). In this context, recycling raw coal waste can be found to be a workable way to make concrete aggregates. Karimaei et al.^12^ tested the green concrete having untreated coal wastes to investigate its mechanical properties. The results indicated that the untreated coal waste could be reused again in concrete production as the substitution of gravel and sand resulted in an average increase in flexural and compressive strength of 5–8% and 3–7%, respectively.

Further validating coal waste’s potential, investigations demonstrated that coal could be a suitable filler in reinforced concrete. It is recognized as a practical solution for producing coarse or fine aggregates. To examine the impact of coal waste as a fine and coarse aggregate replacement on the properties of steel fibers and polypropylene fibers reinforced concrete, Karimipour^13^ tested two groups of mixtures. In the first group, the natural coarse aggregate was substituted with the coal coarse aggregate. In the other group, the natural fine aggregate was substituted with the coal fine aggregate with different replacement percentages. The results indicated that 10% of natural aggregates (coarse and fine) could be substituted by coal aggregates when 2% of fibers (steel fibers and polypropylene fibers) were used, with no reduction in the mechanical properties of reinforced concrete. Amiri et al.^14^ carried out an experimental study to determine the optimal concrete mix proportions produced with coal waste and examine the effect of four variables on the mechanical properties of concrete. These variables included water-to-cement ratio, cement content, gravel volume, and coal waste. The results showed that the optimum values of the water-to-cement ratio, cement content, gravel volume, and coal waste are 0.44, 304 kg/m^3^, 954 kg/m^3^, and 36.95 kg/m^3^, respectively. In addition to concrete’s compressive strength, the water absorption is about 46.30 MPa and 7.41%, respectively. Moreover, Mijan et al. (2024) applied a study on lightweight expanded clay aggregate (LECA), Pumice, and coal waste. Here, LECA was used as a coarse aggregate, and Pumice was used as a fine aggregate, while coal waste was used as a partial replacement for cement to show the impact of these two lightweight materials on SLWC. The results show that using Pumice as a partial replacement for sand and LECA as a coarse aggregate showed significant results and can be used for the production of SLWC. Also, adding coal waste as a partial replacement for cement improves the properties of SLWC.

Despite the efforts of researchers to enhance the mechanical and thermal properties of lightweight concrete, limited attention is paid to the utilization of metallurgical coal (MC) refuse material as a replacement for natural coarse aggregate in SLWC. Despite its low unit weight, thermal stability, and high porosity, which make it a lightweight classification, its availability and potential advantages as a lightweight aggregate. Accordingly, this study aims to use MC to develop SLWC with reduced density, enhanced mechanical properties, and improved sustainability compared to NWA. Thus, it is essential to optimize the material composition to achieve a cost-effective and eco-friendly alternative that maintains structural integrity while reducing environmental impact and construction costs. To this end, an experimental study was carried out to determine whether the MC was a suitable filler for the concrete. Crushed aggregates from MC have partially replaced normal coarse aggregate and are carried out with five variations, namely 0%, 25%, 50%, 75%, and 100% of the lightweight aggregate (LWA), where 0% of LWA functioned as a control mix. To examine the benefits of the MC as a coarse aggregate and its influence on reinforced concrete, tests were done by examining the lightweight hardened concrete. The experimental program for hardened concrete with its five variations includes evaluating the unit weight, compressive strength, flexural strength, modulus of elasticity, and the influence of elevated high temperature on the hardened concrete. The modulus of elasticity was calculated across four international codes, the Egyptian Code of Practice (ECP)^15^, American Concrete Institute (ACI)^16^, Indian Standards (IS)^17^, and Eurocode^18^, to assess the experimental and analytical performance of SLWC using MC. Building on the historical and modern applications of SLWC, this study examines the hypotheses that elevated MC content will decrease concrete density, affect mechanical and thermal performance, and provide potential economic and sustainability advantages, with five MC replacement levels (0%, 25%, 50%, 75%, 100%) examined experimentally. The investigation not only addresses the structural applicability of MC-based SLWC but also contributes to the state-of-the-art for sustainable reuse of industrial by-products in construction.

## Materials and concrete mixtures

### Materials

**Fig. 2:**
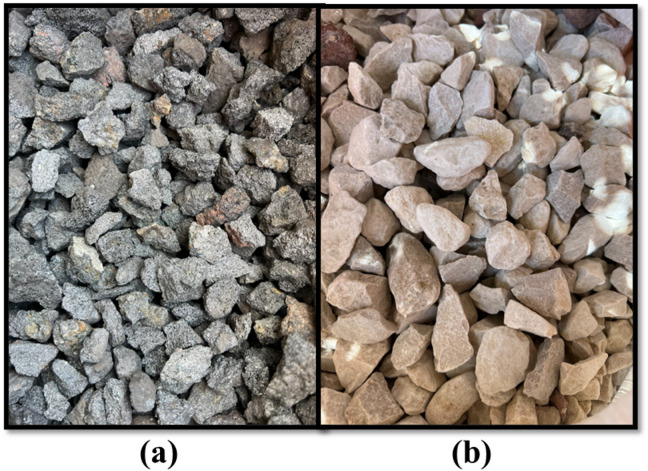
Types of coarse aggregate considered in the study: (a) Metallurgical coal (MC); (b) normal-weight aggregate (NWA).

**Fig. 3:**
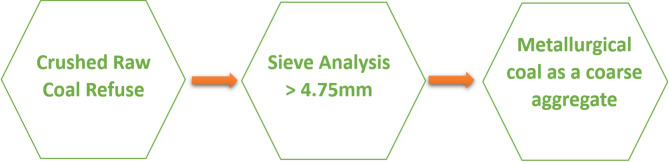
Crushed raw coal to form lightweight coarse aggregate.

Gathering research materials is the first step in the research process. Among these materials are fine aggregate, coarse aggregate, and cement. The fine aggregate used is natural clean sand derived from Katameya Road quarries. The water used in all mixtures was potable water from the water supply system. It was fresh and free from any impurities, organic materials, or any terminated solids that would affect the quality and performance of concrete during the tests. The coarse aggregate consists of two types: normal aggregate, which is derived from Ataka mountain in Suez Governorate, and MC lightweight aggregate, which is derived from Bahariya Oasis road quarries, as it achieves high mechanical properties (Fig. 2). We partially replaced normal coarse aggregate with crushed aggregates from MC based on five variations: 25%, 50%, 75%, and 100% MC. These wide ranges of replacement are crucial to determine the threshold at which MC remains structurally viable^19^. In addition, 0% MC functioned as a control mix to allow a clear comparison with the normal weight concrete. 25% and 50% MC represent partial replacement levels commonly used to identify the optimum balance between acceptable mechanical properties and reduction in the density. These percentages are realistic and applicable in practice for structural lightweight concrete. 75% and 100% MC identify the upper limits of MC usage, while giving a full replacement feasibility, helping in understanding the MC performance boundaries. The maximum nominal sizes of fine aggregate and coarse aggregate are 4.75 mm and 37.5 mm, respectively. The cement is ordinary Portland cement CEM1 grade 42.5 N with properties shown in Table 1. The performance of sand, NWA, and LWA was detected through mechanical properties such as sieve analysis, specific gravity, bulk density, and water absorption tests. Figure 3 shows the process of producing lightweight MC as coarse aggregate from crushed MC.

**Table 1:** Properties of ordinary portland cement.

Properties	Values
Fineness	3330 cm^2^/g
Specific gravity	3.15
Initial setting time	60 min
Final setting time	240 min

### Concrete mix design

Concrete mixes are designed based on the American Concrete Institute (ACI) with standard practice for selecting proportions of concrete^20^. All specimens in the experimental program were prepared, mixed, and cured under identical, rigorously controlled laboratory conditions. No curing, mixing time, or temperature exposure was made variable at any replacement level, all procedures followed standard ASTM C192, ensuring that only the replacement level represented a variable in the experimental design. All specimens were cured under water in a curing basin at 23 ± 2 °C. The mixing time of the concrete mixture was 3 min of initial mixing, a rest for 3 min, and then final mixing for 2 min. The temperature exposure of both materials during mixing and fresh concrete was typically in the range of (20–30 °C). Table 2 summarizes the baseline parameters following ACI standards practice for selecting proportions in structural lightweight concrete (SLWC) mix design. These values guided the development of all mixtures, with variations applied for metallurgical coal (MC) replacement levels.

**Table 2:** Mix design parameters.

Parameters	Designed values	Units
Choice of slump	80–100	mm
Maximum aggregate size selection	25	mm
Mixing water selection	195	kg/m^3^
Air content selection	1.5%	-
Water-cement ratio	0.46	-
Cement content	423.91	kg/m^3^
Coarse aggregate content	According to the mixture variation	-
Fine aggregate content	According to the mixture variation	-
Adjustments for aggregate moisture	According to the type of aggregates	-

### Mixed proportions of concrete mixtures

In this paper, MC has partially replaced the normal coarse aggregate by weight with five different variations 25%, 50%, 75%, and 100%, and there is one mixture that acts as a control mix. Table 3 shows these variations with the mixed proportions of concrete main components (cement, fine aggregate, coarse aggregate, and nominal water). Casting fresh concrete using MC with different variations is the same as casting the control mixture of 0% of MC, except for one difference. This difference is due to the water absorption of MC, which could cause water shortage during casting and affect the workability of fresh concrete, so it must be considered in the mixtures. To provide uniform workability across mixes, the high-water absorption of MC has been compensated for during mix design. The water absorption of MC was experimentally found to be 7.03%. An extra amount of mixing water was computed for each mixture by multiplying 7.03% by the weight of MC used in that mixture. This extra amount was subsequently added to the nominal mixing water.

**Table 3:** Ratio of mixed proportions of concrete by weight.

Mixture of concrete	Ratio of mixed proportions C:FA:NWA:MC:W	Cement (kg/m^3^)	FA (kg/m^3^)	NWA (kg/m^3^)	MC (kg/m^3^)	Nominal water (kg/m^3^)
0% MC	1:1.74:2.4:0:0.46	424	738	1018	0	195
25% MC	1:1.29:1.5:0.5:0.46	424	547.55	640.3	213.5	195
50% MC	1:1.19:0.81:0.81:0.46	424	505.69	345	345	195
75% MC	1:1.4:0.31:0.93:0.46	424	592.1	131.25	393.75	195
100% MC	1:1.9:0:0.85:0.46	424	806.3	0	359.5	195

## Experiments

**Fig. 4:**
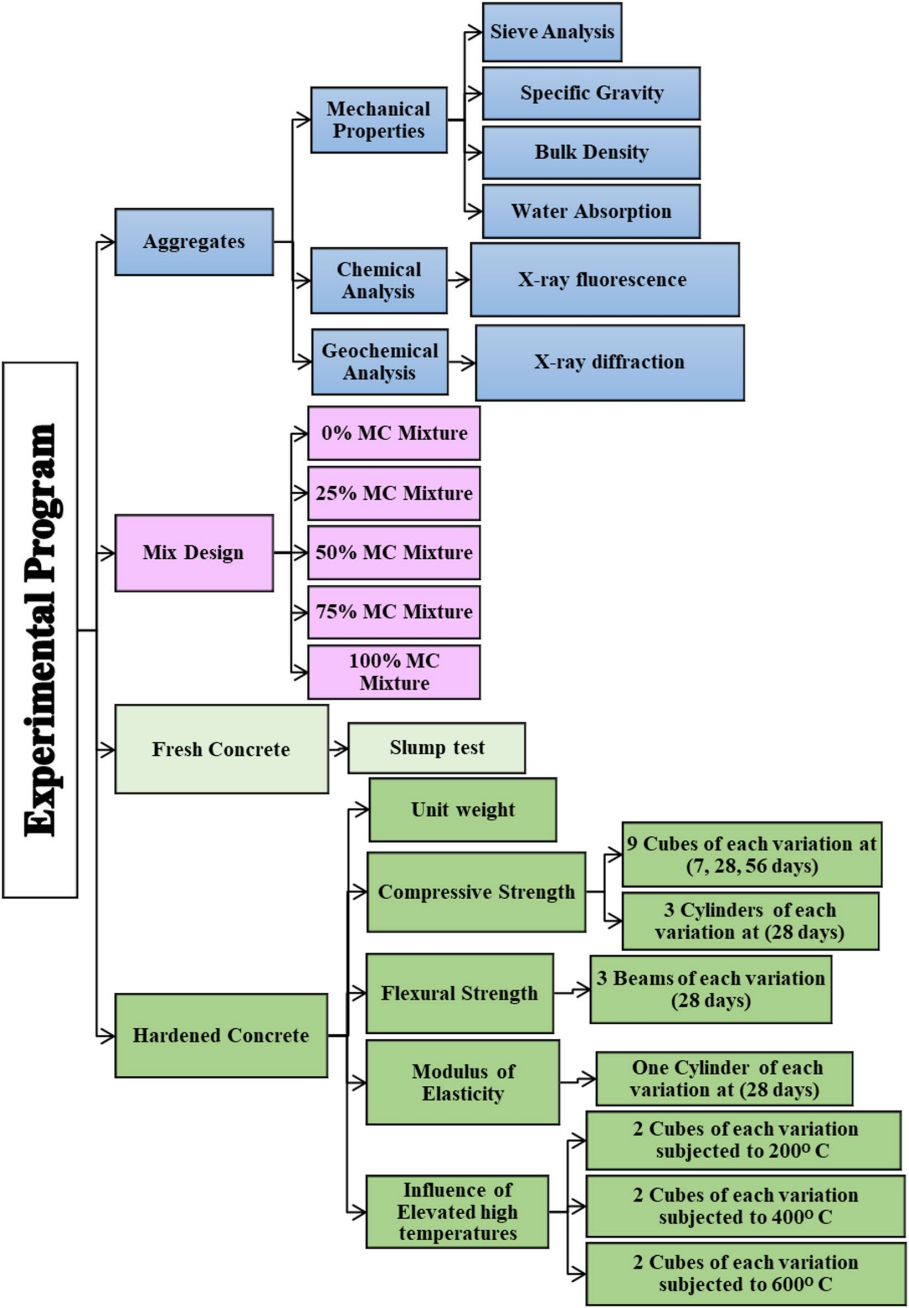
Experimental program of the study.

Figure 4 shows the experimental program of SLWC mixtures done in this paper to determine the performance of SLWC using MC as a coarse aggregate used in concrete. By conducting these experimental programs, the overall behavior of the concrete using MC as a lightweight coarse aggregate is established, and the feasibility of using it as a coarse aggregate in structural demands and applications is demonstrated.

### Aggregates tests

The experimental program shows many tests conducted on the aggregates to test their mechanical properties by testing the sieve analysis, specific gravity, bulk density, and water absorption for all types of aggregates, either fine aggregate or coarse aggregate. The sieve analysis test was conducted to determine the particle size distribution of aggregates and whether the aggregates meet the required aim. The test procedure was setting the standard sieves for fine aggregate and coarse aggregate with varying mesh sizes. Then, the weighted sample of both aggregates was placed on the sieves and shaken. Finally, the retained percentage of aggregates was weighed to detect the percentage of passing aggregates to evaluate the aggregate gradation. Secondly, the specific gravity was tested to ensure the concrete’s performance regarding its strength and durability. The test was conducted by weighing the samples of both aggregates before and after submerging them in water for 24 h to ensure complete saturation. Subsequently, the volume was calculated to determine the specific gravity of each type. Thirdly, the bulk density of both types was evaluated to determine the amount of normal-weight aggregate (NWA) and lightweight aggregate used. A sample of aggregates was filled in a container with a known volume in three layers, and compaction (25 times for each layer) was done by using a metallic rod for both sand and coarse aggregate, then bulk density was evaluated. Finally, the water absorption test was conducted to evaluate the absorption percentage of each type of aggregate. A weighed sample of aggregates was submerged in water for 24 h to ensure full saturation after being dried in an oven. Then, the chemical composition of the MC was detected by using X-ray fluorescence (XRF). It is a technique used to detect the components of the MC and to detect the percentage of the oxide content inside the MC. According to the^21^ and^22^, the test was conducted, and the results were reported. The environmental conditions when the test was conducted were detected. The degree temperature was 22 °C ± 1 °C and the relative humidity was 44% ± 1%. Finally, the geochemical analysis of the MC was detected by using X-ray diffraction (XRD). It is a technique used to determine the mineralogical composition of a sample of the MC. The sample used was in a powder form of MC. Copper is used as an anode material in the X-ray tube’s anode, which is critical in X-ray generation as it ensures the diffraction patterns and accurate analysis. The test was conducted, and the results were reported. The environmental conditions when the test was conducted were detected. The degree temperature was 20.5 °C ± 1 °C and the relative humidity was 24% ± 1%.

### Fresh concrete tests

To guarantee the performance and quality of fresh concrete, a slump test was performed to identify the workability using the slump test for all concrete mixtures with different variations^23^. The slump test was conducted using the Abrams cone, whose dimensions are (300 mm height, 100 mm top diameter, 200 mm bottom diameter). It was experimentally investigated by filling the cone, then raising it vertically and slowly, allowing the concrete to flow freely under its own weight, and subsequently measuring the slump value.

### Hardened concrete tests

The performance of NWA and SLWC was detected through mechanical tests, as compressive srength tests for cubes^24^ and cylinders^25^, unit weight^26^, modulus of elasticity^15, 17, 18^, and flexural strength of concrete^27^. In addition, the durability test specimens were subjected to different heat temperatures to determine the effect of the elevated temperature on the concrete.

As for the hardened concrete, which was cast by five variations for partial replacement of NWA, which are 0%, 25%, 50%, 75%, and 100% MC. The variation of 0% of MC is considered as NWA, and variations 25%, 50%, 75%, and 100% MC aggregate are considered as SLWC. The experimental program tests the mechanical properties of hardened concrete using a universal testing machine by testing its compressive strength in two ways. The first way is the concrete casting of 9 cubes of each variation with dimensions (150 mm × 150 mm × 150 mm) and testing these cubes after 7 days, 28 days, and 56 days. The other way is casting 3 concrete cylinders of each variation with a diameter of 150 mm and a height of 300 mm, and testing them after 28 days. Subsequently, the concrete’s flexural strength is tested by casting 3 beams of each variation with dimensions (100 mm × 100 mm × 700 mm) and testing these beams after 28 days by loading them with the four-point load to determine the concrete’s flexural strength.

An evaluation of the concrete modulus of elasticity was obtained by calculating different codes. The calculation of the modulus of elasticity in this study of SLWC and NWC was conducted, and a comparison between the modulus of elasticity based on these codes was made.

According to the ECP, the modulus of elasticity is calculated using Eq. (1), which detects the relationship between the compressive strength and the modulus of elasticity as specified by the code.

1 $$E_{c} = 4400\sqrt {f_{cu} }$$

where $E_{c}$ is the modulus of elasticity in MPa and $f_{cu}$ is the characteristic compressive strength of concrete cubes in MPa. The second calculation for the modulus of elasticity shown in Eq. (2) was done according to the ACI, taking into consideration its compressive strength of conrete and its unit weight as specified by the code.

2 $$E_{c} = 0.043w_{c}^{1.5} \sqrt {f^{\prime}_{c} }$$

where w_c_ is the unit weight of concrete in kg/m^3^ and $f^{\prime}_{c}$ is the characteristic compressive strength of concrete cylinders in MPa. The third calculation for the modulus of elasticity shown in Eq. (3) was done according to the IS, which detects the relationship between the cylinder’s compressive strength and the modulus of elasticity of concrete as specified by the code.

3 $$E_{c} = 5000\sqrt {f_{ck} }$$

where $f_{ck}$ is the characteristic compressive strength of concrete cylinders in MPa. The fourth calculation for the modulus of elasticity shown in Eq. (4) was done according to the Eurocode, which detects the relationship between the cylinder’s compressive strength and the modulus of elasticity of concrete as specified by the code.

4 $$E_{c} = 22000\left[ {\frac{{f_{cm} }}{10}} \right]^{0.3}$$

where $f_{cm}$ is the average compressive strength of the cylinder in MPa.

The final test regarding hardened concrete is the performance and strength of the concrete after 28 days of curing, which is a durability test of SLWC. Under the influence of elevated high temperatures, many chemical and physical reactions occur in the concrete as dehydration of cement paste and water loss of the concrete mix^28, 29^. Six cubes of these three variations (0% MC, 25% MC, 50% MC were subjected to elevated high temperatures. The six cubes of each variation were divided into three groups,each group consisted of two cubes of each variation. The groups were subjected to a thermal range of 200 °C, 400 °C, and 600 °C. These exposure temperatures were selected to reflect different thermal conditions relevant to the fire performance of structural concrete. The lower temperature, which is 200 °C, indicated the first signs of moisture migration and the early microcracks. The 400 °C is an intermediate level where degradation of cement paste and aggregate paste bond is observed. The 600 °C represents a major fire scenario in which the load-bearing capacity is still practical. These distinct levels correspond to temperature intervals commonly used in earlier experimental studies on normal-weight and lightweight concretes at high temperatures^30, 31, 32^. The heating duration of each group at each temperature was 2 h. To maintain more of the concrete’s strength, slow cooling can minimize the chance of cracks that have occurred and thermal gradients^33^. Therefore, after heating the concrete cubes, the specimens were left in the open air for 24 h to cool down naturally before testing.

## Results and discussion

According to the experimental program stated before, the mechanical properties of both types of aggregates were detected. The chemical composition and geochemical analysis of MC were stated. The mechanical properties of the fresh concrete and hardened concrete of all concrete mixtures were detected.

### Aggregates

**Fig. 5:**
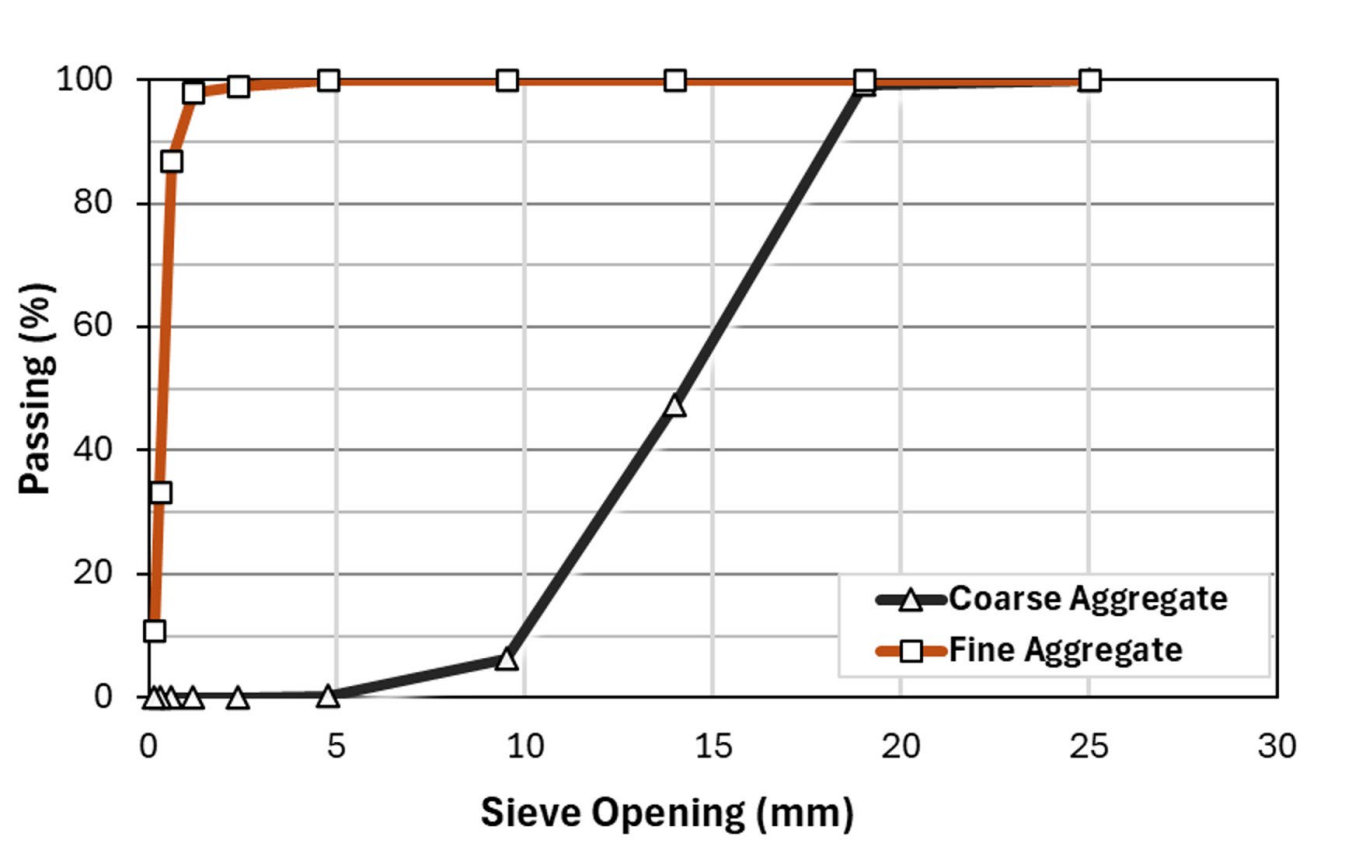
Gradation curve of coarse aggregates (MC or natural aggregates) and fine aggregates.

Figure 5 indicates the coarse aggregate, which is the normal aggregate, and the MC gradation curve, and also the fine aggregate gradation curve resulting from the sieve analysis test conducted separately, which determines the particle size distribution of fine and coarse aggregates. According to the results presented in Table 4, the specific gravity of sand is 2.55, the NWA is 2.61, and the MC aggregate is 1.015. Based on these findings, the specific gravity of normal-weight coarse aggregate is higher than that of MC and sand, as the NWA consists of denser materials and has lower porosity.

**Table 4:** Results of aggregate tests.

Properties	FA	NWA	MC	Units	Standards
Specific gravity	2.55	2.61	1.02	-	^34^
Water absorption	1	2.1	7.03	%	^35^
Bulk density	1765	1520	501.33	kg/m^3^	^36^

Table 4 shows the test results of the fine aggregate, coarse aggregate (NWA), and MC. The results indicated that the bulk density of NWA is higher than that of the MC aggregate. The reason is that the particle size of the NWA is more angular and compact, which reduces its voids, resulting in a higher weight than the MC. Moreover, the results of the water absorption test, the NWA is 2.1%, 7.03% for MC, and 1% fine aggregate. The results stated that the MC aggregate has a higher absorption percentage as it has higher porosity.

The oxide composition of MC is assessed using XRF analysis. According to ^21, 22^, the results are shown in Table 5. The results indicated that the major oxides present in the MC were silicon dioxide (SiO_2_), aluminum oxide (Al_2_O_3_), and sulfur trioxide (SO_3_). Traces of other oxides were present in the MC chemical composition, and a large content of loss of ignition (LOI) as volatile matter and the decomposition of carbonates, which decomposed at high temperatures, causing the (CO_2_) and sulfur compounds as (SO_3_), contributing to the mass loss during heating.

**Table 5:** Oxide content % of MC.

Constituents (oxide content)	Values (%)
SiO_2_	13.56
Al_2_O_3_	7.38
Fe_2_O_3_	6.89
CaO	2.88
MgO	0.56
Na_2_O	0.25
K_2_O	0.68
SO_3_	9.55
TiO_2_	0.71
P_2_O_5_	0.28
MnO	0.07
Cr_2_O_3_	0.14
Cl-	0.15
Loss of Ignition	56.90

**Fig. 6:**
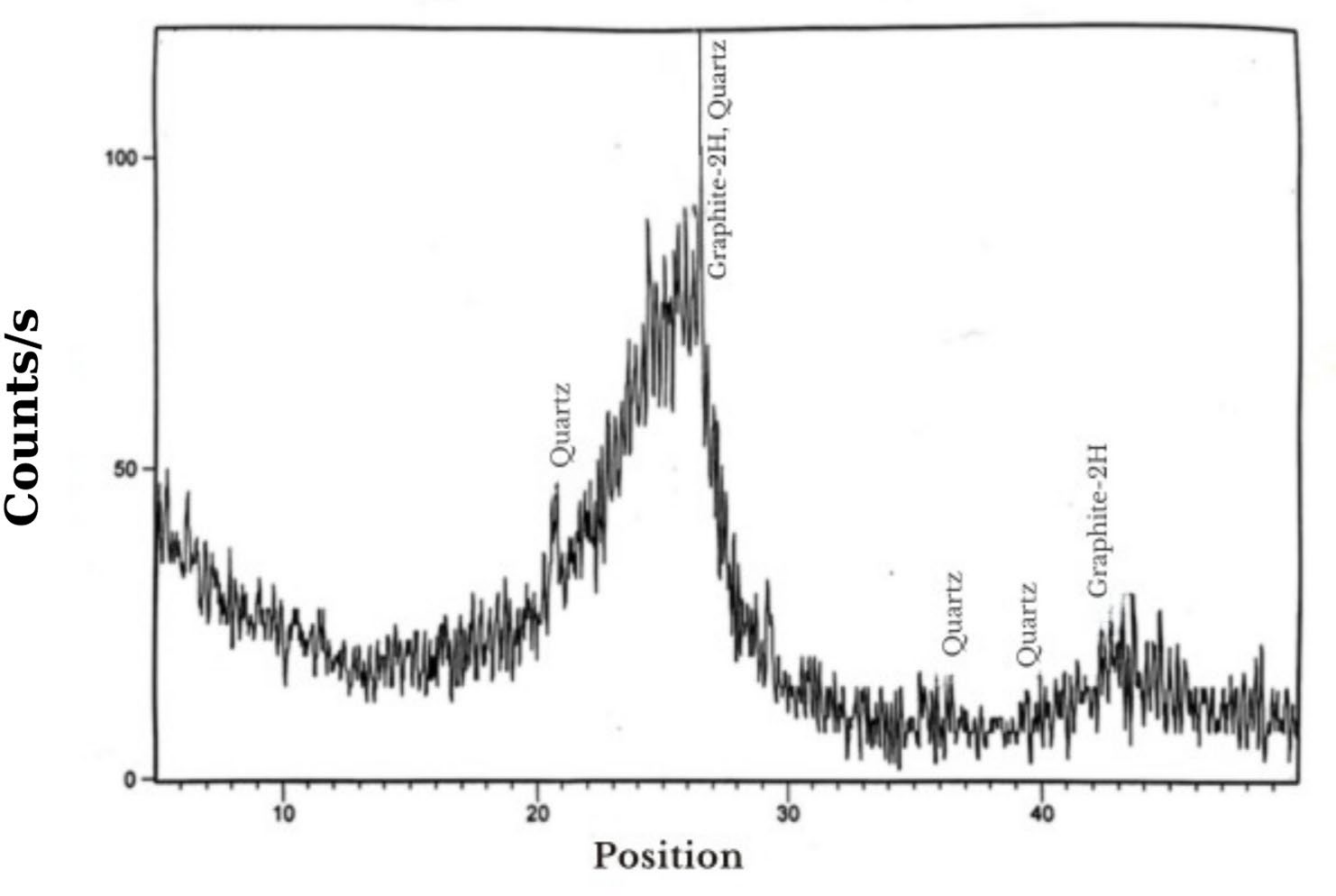
XRD pattern of the MC.

X-ray diffraction is utilized to examine the mineralogical content of a sample of the MC. The most probable phases were identified utilizing the PANalytical computer certification tool, which is X’pert High Score software 2006. Table 6 shows the compounds and the mineral compositions of the MC. These compounds were Graphite-2H and Quartz. As appeared from the XRD pattern, the MC was composed mainly of Graphite (C) and traces of Quartz (SiO_2_) minerals. The pattern shows a large amorphous hump with noises appearing in 2ϴ between 20° and 30°, as shown in Fig. 6.

**Table 6:** Compounds content of MC.

Compound name	Chemical formula
Graphite-2H	C
Quartz	SiO_2_

### Fresh concrete

**Fig. 7:**
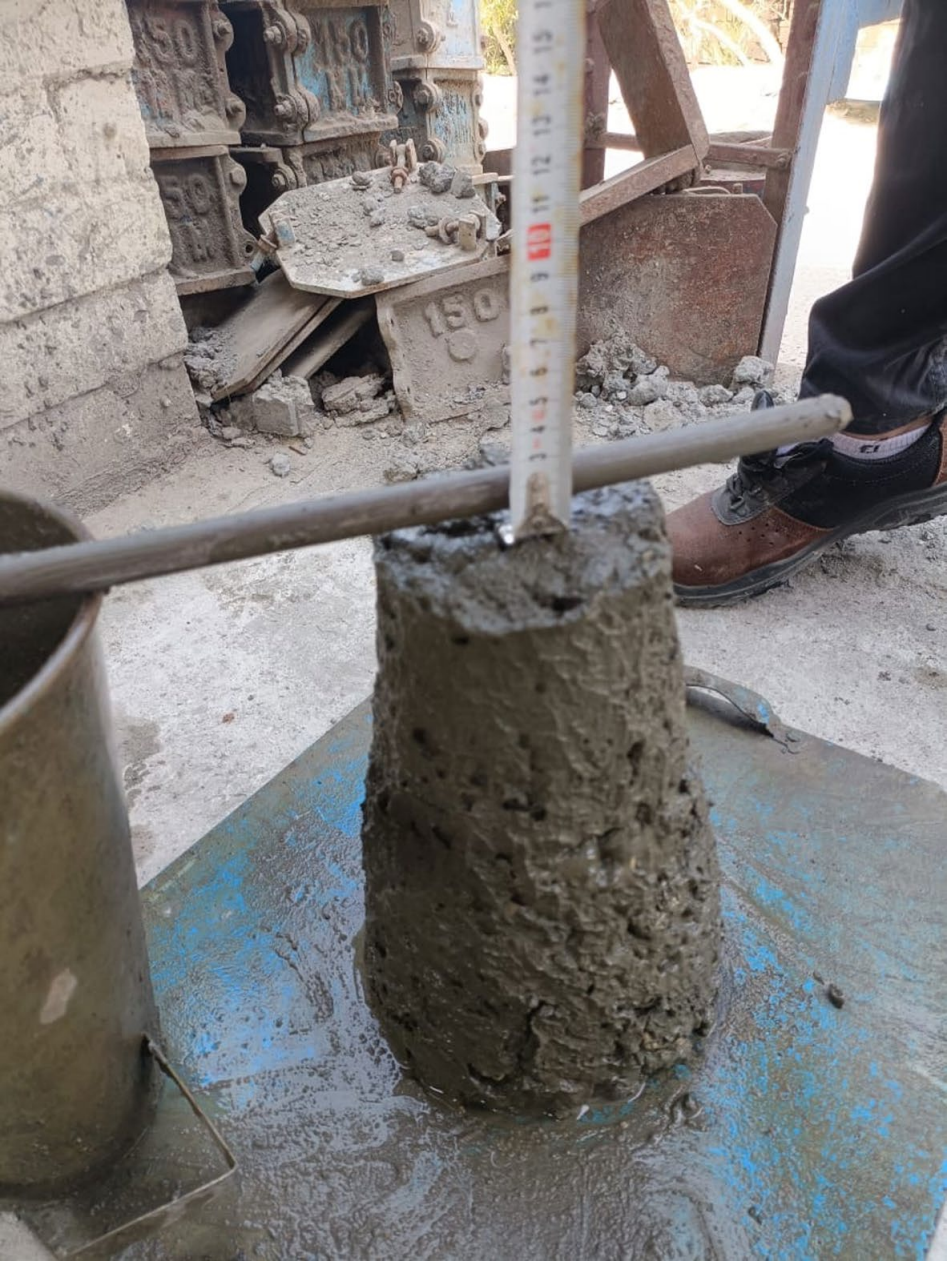
Slump test of concrete.

The slump test was demonstrated to detect the workability of NWA and SLWC, as shown in Fig. 7. The results show that utilizing MC as an aggregate reduces the workability of concrete. The results regarding the effect of replacing MC with NWA on slump values are shown in Table 7. These results show that the higher the NWA replacement percentage, the lower the slump value. This means that the water absorption of MC due to its voids decreases the slump value of fresh concrete and has a detrimental influence on its workability. The discrepancy between the target slump (80–100 mm) and measured values (20–40 mm) is due to the high water absorption of the MC aggregate (7.03%). Although additional water was added to compensate for absorption, the aggregate’s porous nature still reduced overall workability. This observation is supported by Aboutaybi and Yang^37^, who found that coal mine waste absorbs more water due to finer particle size, resulting in an 18% decrease in slump. Similarly, Shin et al.^38^ found that the porous nature of LWA leads to higher loss of slump and poorer workability of concrete.

**Table 7:** Slump test on concrete.

Mixture of concrete	Slump value (mm)
0% MC	40
25% MC	30
50% MC	25
75% MC	20
100% MC	20

### Hardened concrete test results

#### Compressive strength

**Fig. 8:**
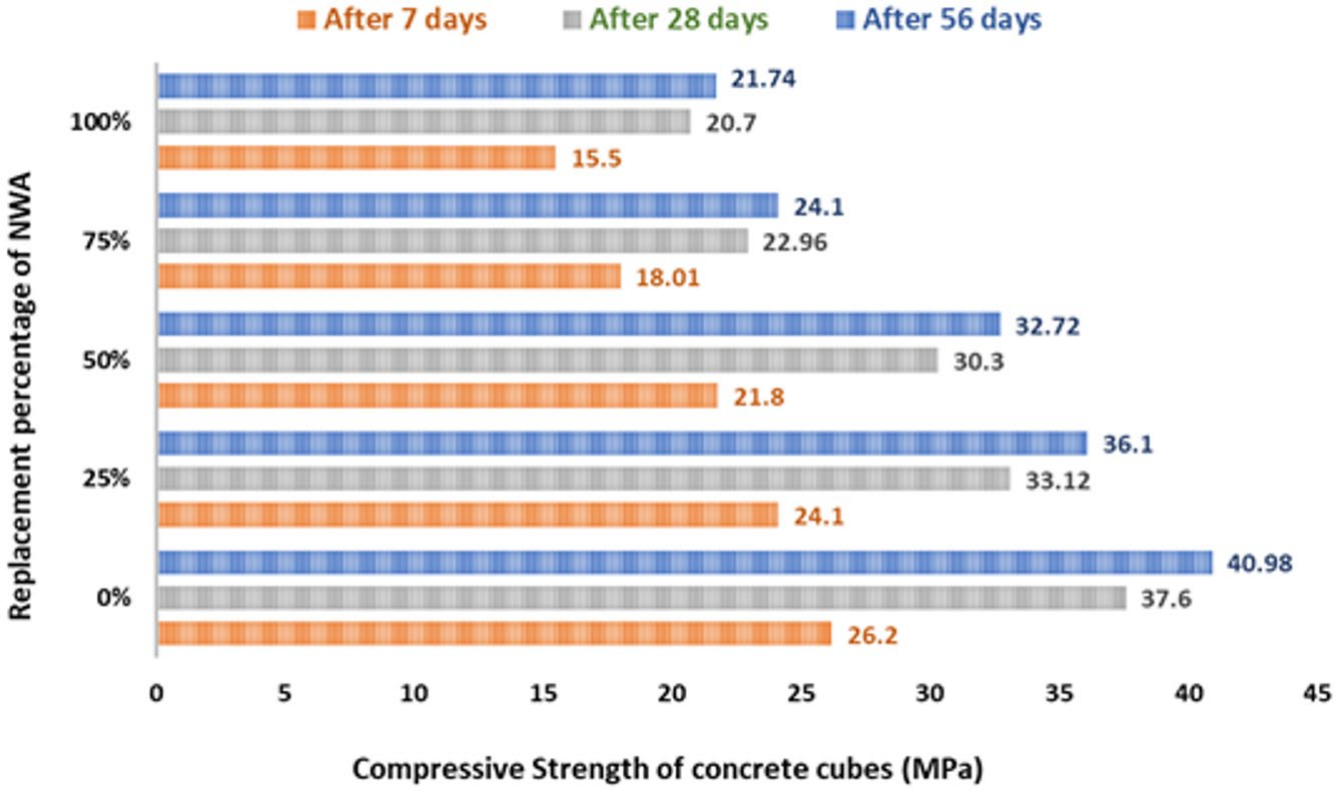
Compressive strength of concrete cubes during 7, 28, 56 days.

This section summarizes the findings and contributions made in the study according to the experimental program mentioned before. Based on the findings, it’s clear that increasing the MC content in concrete mixtures resulted in a considerable reduction in the compressive strength of the concrete. Figure 8 shows the compressive strength values of the SLWC cubes after 7 days of the five different replacement variations. These values decreased from 26.2 MPa to 15.5 MPa at 0% and 100% of replacement, respectively. In addition, the compressive strength values of the SLWC cubes after 28 days. These values decreased from 37.2 MPa to 20.7 MPa at 0% and 100% of replacement, respectively. Furthermore, the compressive strength values of the SLWC cubes after 56 days of the five different replacement variations. These values decreased from 40.98 MPa to 21.74 MPa at 0% and 100% of replacement, respectively. Accordingly, the compressive strength decreases significantly with increasing MC replacement at all curing ages (7, 28, and 56 days) and is almost increased with the longer the concrete is aged in all mixtures.

**Fig. 9:**
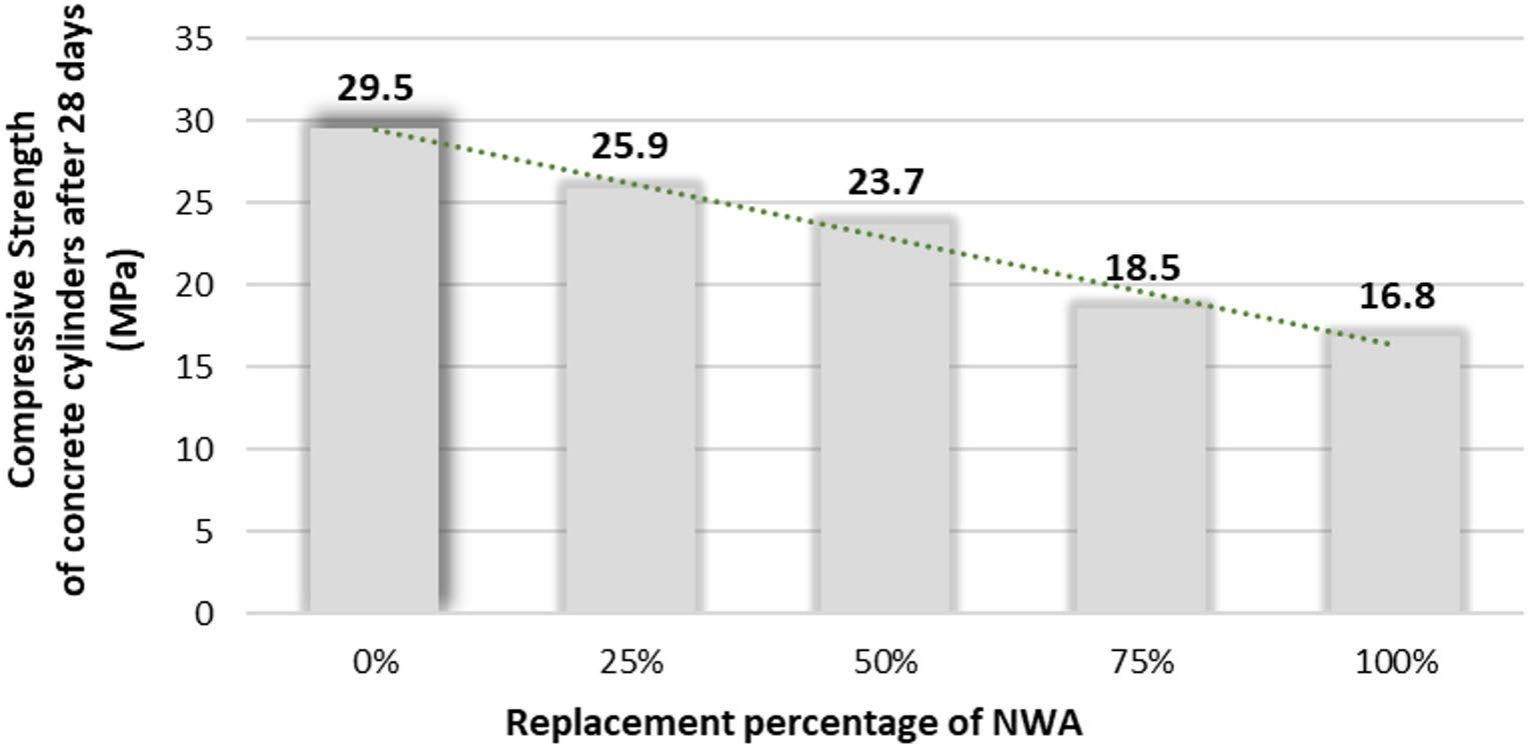
Compressive strength of concrete cylinders during 28 days.

According to the experimental program, the compressive strength was also measured by concrete cylinders, resulting in a reduction in the compressive strength from 29.5 to 16.80 MPa at 0% and 100% of replacement, respectively, as shown in Fig. 9. There are different thresholds concerning the SLWC according to ACI 213R-87. These thresholds stated that the compressive strength of SLWC cylinders must be more than 17.2 MPa. Accordingly, in this study, the rate of strength of all replacement variations was satisfied with the specified compressive strength thresholds except for the 100% MC. Therefore, 100% MC could not be considered as an SLWC, although it is a lightweight concrete. The fundamental explanation for the reduction in compressive strength with increasing metallurgical coal (MC) aggregate replacement is mainly due to the weak and porous nature of MC compared to natural weight aggregates (NWA). MC increases overall concrete porosity and weakens the interfacial transition zone (ITZ) due to the high-water absorption and surface roughness contributing to the formation of porous and less dense ITZ. Therefore, it leads to inefficient load transfer and more microcracking as the ITZ is already considered the weakest region in the cementitious material. Although MC is lightweight and usable in SLWC, it results in strength loss because of these microstructural deficiencies. This is substantiated by refs.^39,40,^ who showed that coal-derived aggregates lower the compressive strength of concrete and adversely impact ITZ quality, prompting microcracking and reduced bonding strength.

#### Unit-weight of concrete

There are different thresholds concerning the SLWC according to ACI 213R-87. These thresholds demonstrated that the unit weight must be less than 1850 kg/m3. The unit weight of concrete mixtures is computed and presented in Table 8, it’s considered that the unit weight decreases as the percentage of MC aggregate increases in the concrete mixtures. The values decreased from 2168 kg/m^3^ for 0% replacement of NWAs to 1642 kg/m^3^ for 100% replacement of NWAs. A popular explanation for this reduction is that the lightweight aggregate bulk density is 501.33 kg/m^3^ whereas the bulk density of NWA is 1520 kg/m^3^. Multiple studies^39, 40, 41^ have shown that replacing natural aggregates with coal by-products and other lightweight aggregates, which have a considerably lower bulk density than natural aggregates, significantly reduces concrete density.

**Table 8:** The unit weight of the concrete mixtures with different variations.

Mixture of concrete	Unit weight (kg/m^3^)
0% MC	2168
25% MC	1877
50% MC	1696
75% MC	1678
100% MC	1642

#### Flexural strength of concrete

**Fig. 10:**
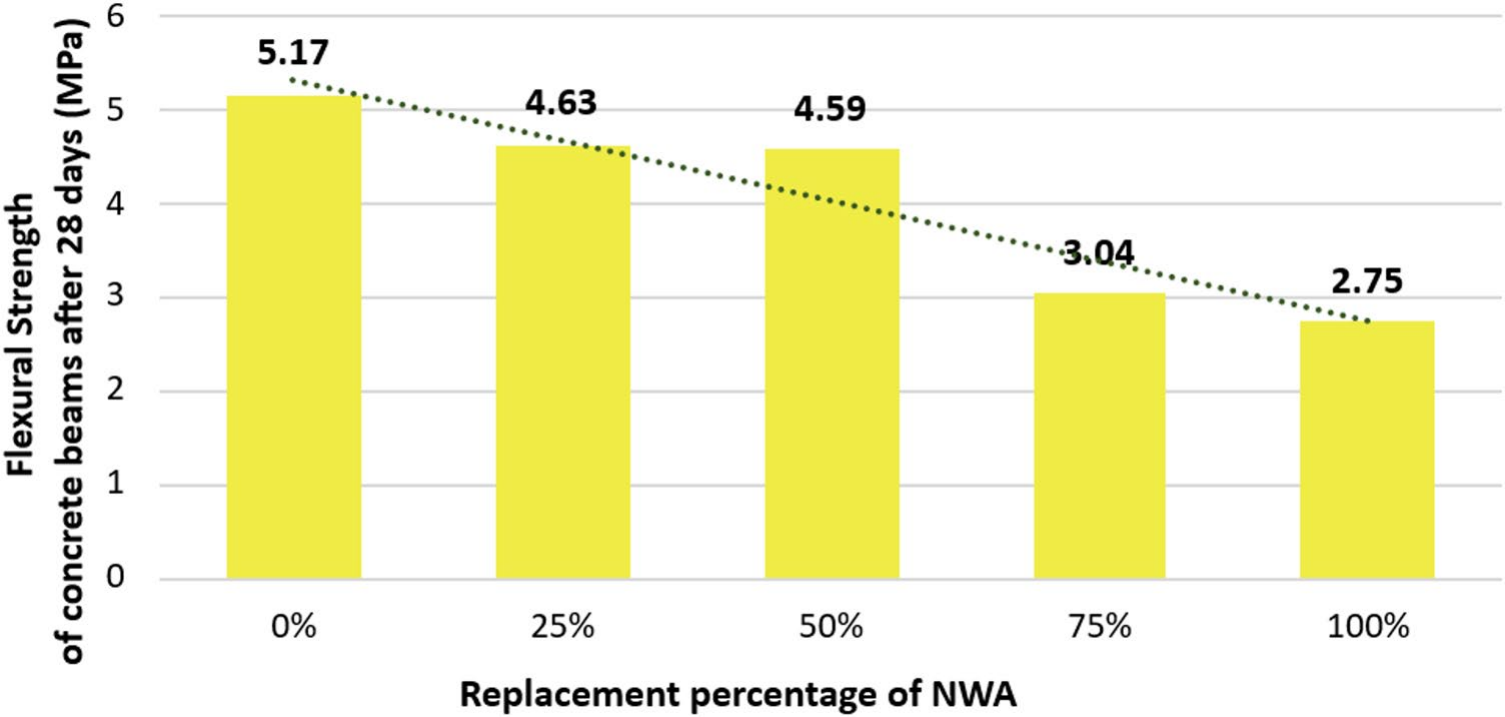
Flexural strength of concrete mixtures after 28 days.

**Fig. 11:**
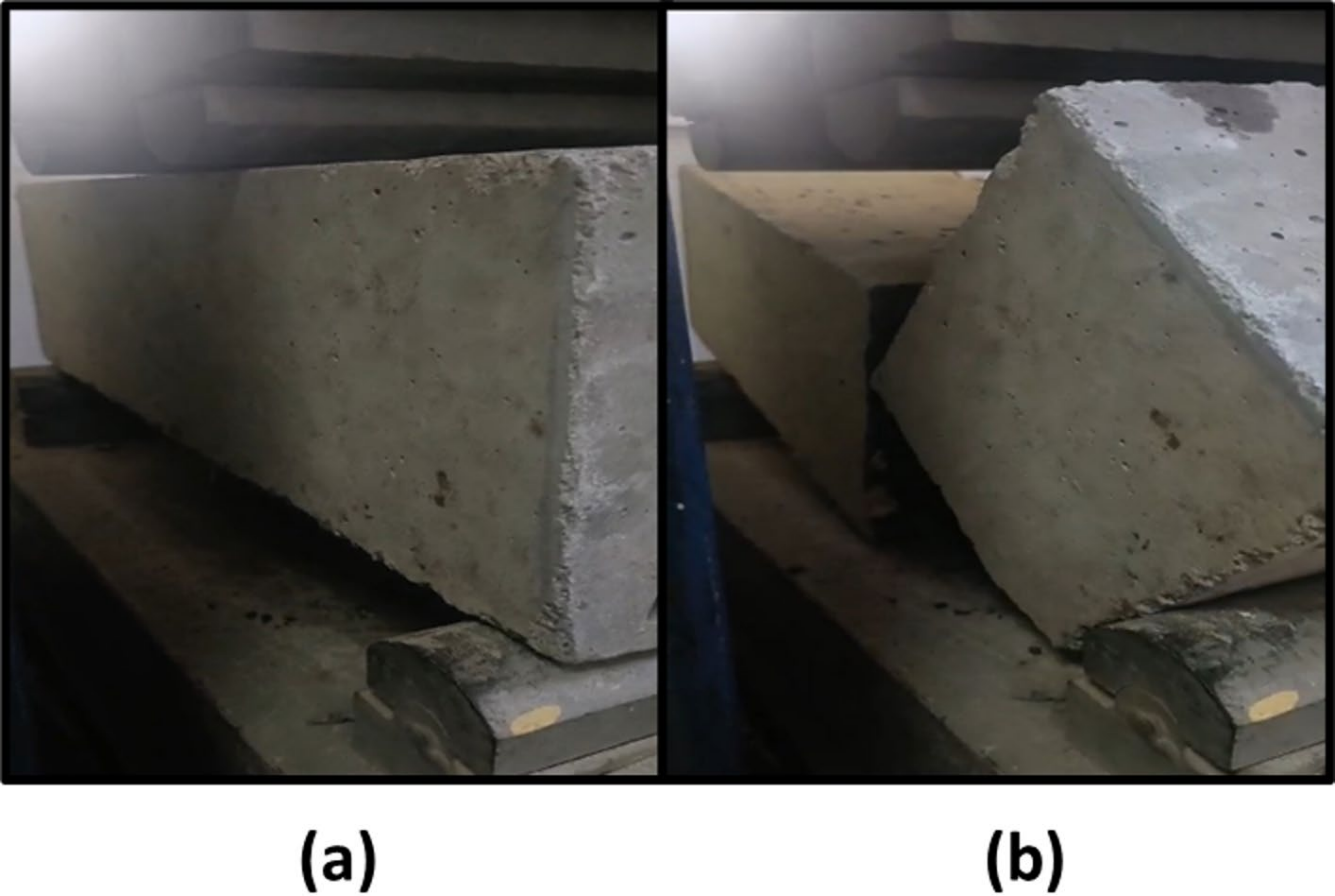
Flexural Strength test: (a) test setup; (b) shape of failure.

Similar to the compressive strength of MC aggregate SLWC with different variations, a higher MC aggregate contribution led to lower flexural strength among all concrete beams with all variations after 28 days. As shown in Fig. 10 the flexural strength is documented to be 5.17 MPa, 4.63 MPa, 4.59 MPa, 3.04 MPa, and 2.75 MPa for the concrete variations (0%, 25%, 50%, 75%, and 100%). Although SLWC provides many benefits, it also leads to the reduction of the flexural strength of concrete. Flexural strength follows the pattern of the compressive strength that with higher MC percentages, the flexural strength leads to consistently lower values. This reduction of the flexural strength values is due to the characteristics of the lightweight aggregate, which has high porosity. The air voids in the MC lead to an increase in the probability that it may crack under the subjection to flexural loads, as shown in Fig. 11. Recent studies have consistently shown that the increase of lightweight aggregates in concrete leads to reduced flexural strength, primarily due to the elevated porosity and greater internal cracking under flexural loads^42,43^.

#### Modulus of elasticity

**Fig. 12:**
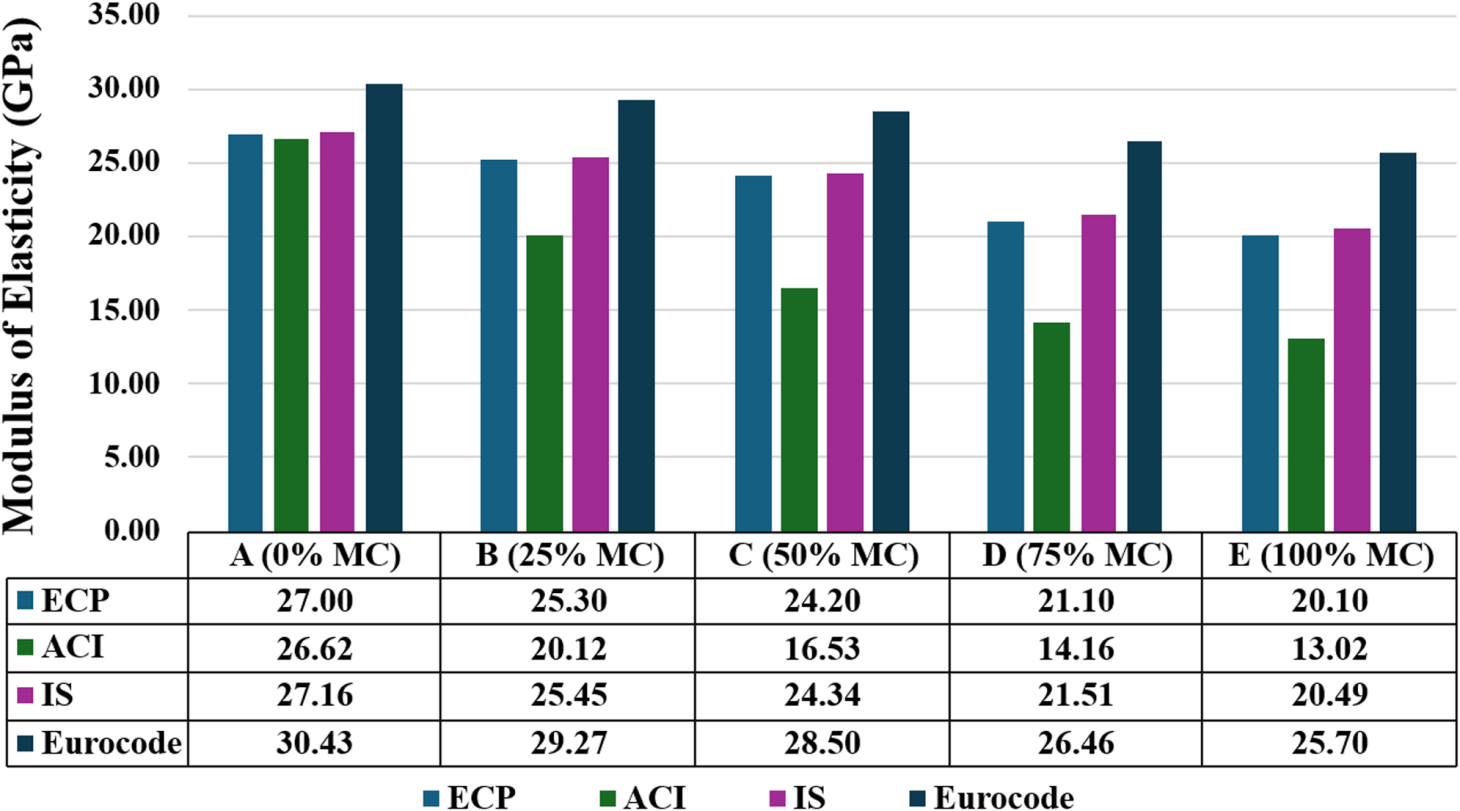
Modulus of elasticity according to ECP code, ACI code, IS code, Eurocode.

The results of the modulus of elasticity are listed in Fig. 12, according to the ECP, ACI, IS, and Eurocode. Across all four design codes, the modulus of elasticity consistently dropped as the replacement level of natural aggregate with MC increased. For instance, ECP values declined from 27.00 GPa at 0% MC to 20.10 GPa at 100%, while ACI showed a steeper fall from 26.62 GPa to just 13.02 GPa. The results show that ECP and IS predict the experimental values well at 0% to 25% MC, but overestimate them as MC rises. The ACI code underpredicts the experimental values, especially with higher replacement levels. Eurocode provides the highest prediction across all mixes. Overall, deviations between codes prediction grows with increasing the MC%. Despite slight variations between codes, it is obvious that as the MC content increases, the modulus of elasticity decreases, and this is the same result by using the four codes. The general trend showed that MC had lower stiffness, highlighting the impact of increased porosity and reduced aggregate strength.

#### Influence of high elevated temperature

**Fig. 13:**
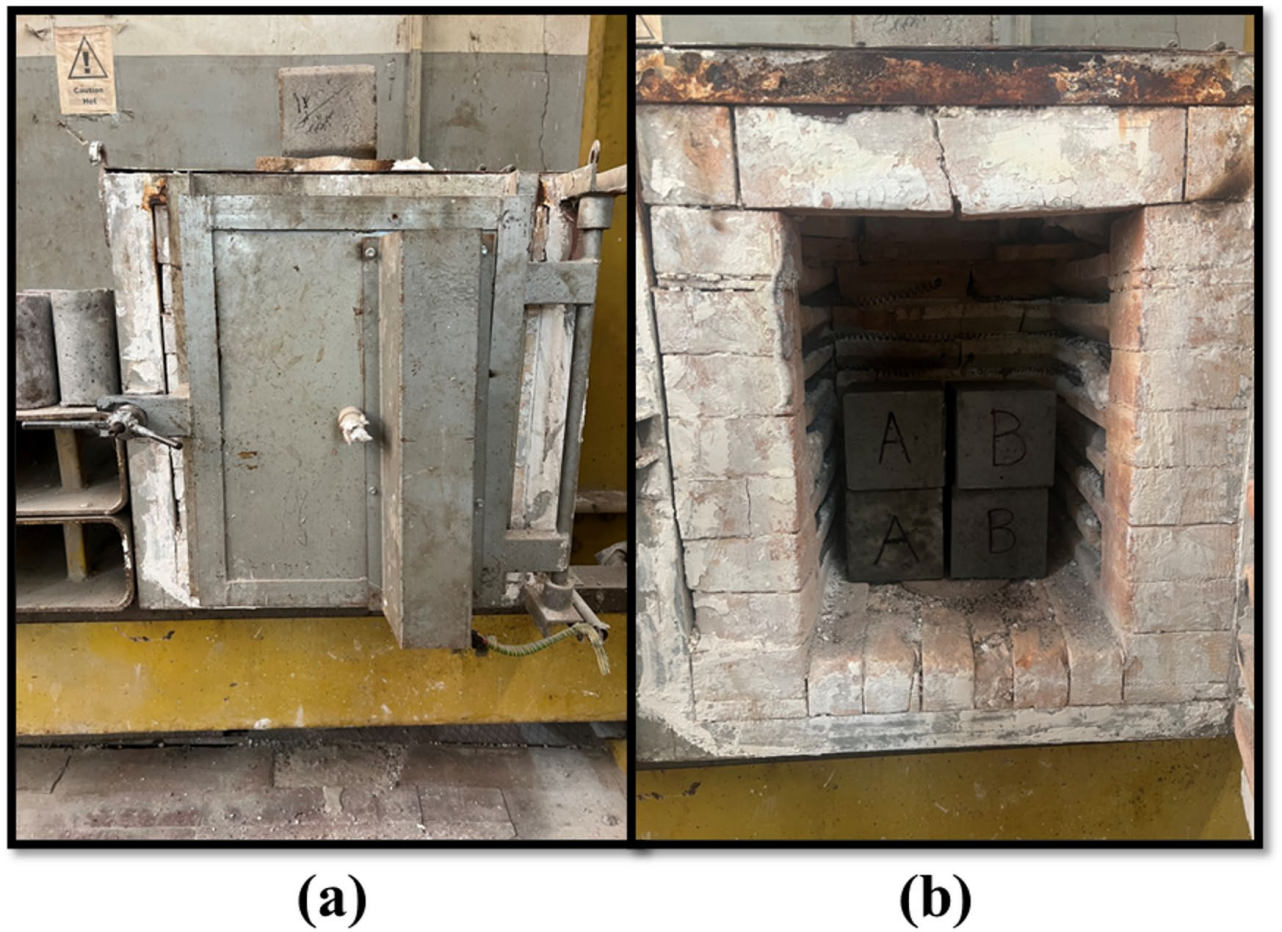
High elevated temperature test; (a) Oven, (b) Samples of concrete mixtures inside the oven.

It was crucial to test eco-friendly concrete for fire resistance and demonstrate the impact of employing MC. According to the performance of concrete, after it was subjected to high temperatures in the chamber furnace. Table 9 displays the residual compressive strength of the concrete mixes after exposure to the elevated high temperature. As mentioned in the experimental program of this study, this test was conducted on only 3 replacement levels (0%, 25%, and 50%) and subjected to 200 °C, 400 °C, and 600 °C, as shown in Fig. 13. The compressive strength of SLWC at 0% replacement of aggregates, whose specimens are named “A”, the values reduced approximately 17.9%, 29.52%, and 40.66% compared to its original strength after exposure to 200 °C, 400 °C, and 600 °C, respectively. In addition, the compressive strength of SLWC at 25% replacement of aggregates, whose specimens are named “B”, the values reduced approximately 17.3%, 24.25%, and 39.28% compared to its original strength after exposure to 200 °C, 400 °C, and 600 °C, respectively. Finally, the compressive strength of SLWC at 50% replacement of aggregates, whose specimens are named “C”, the values reduced approximately 21.25%, 33.68%, and 43.26% compared to its original strength after exposure to 200 °C, 400 °C, and 600 °C, respectively.

**Table 9:** Strength of concrete values after being subjected to elevated temperature.

Specimens	Control	200 °C	400 °C	600 °C
A (0% MC)	37.60	30.86	26.50	22.31
B (25% MC)	33.12	27.39	25.09	20.11
C (50% MC)	30.49	24.01	20.22	17.30

This can be explained by the migration of water from the concrete cubes at high temperatures, leading to rapid moisture evaporation and rapid shrinkage cracks. Moreover, chemical reactions occur at high temperatures, resulting in an impact on the concrete properties and decomposition of lightweight aggregates. The greater reduction in strength at higher MC contents is attributed to the high porosity and thermal instability of the MC aggregate, which promotes vapor-pressure buildup and microcracking. These combined mechanisms accelerate aggregate deterioration at elevated temperatures, resulting in a reduction in the mechanical properties of the mixtures^44, 45^. Despite the reduction of the compressive strength of concrete cubes of all variations under the effect of elevated temperature, the compressive strength of all variations is satisfied with the stated thresholds mentioned before.

The replacement of natural aggregate with metallurgical coal (MC) reduces the unit weight of concrete from 2168 to 1642 kg/m^3^ for 0% and 100% MC, respectively, resulting in a notable reduction in compressive strength, flexural strength, and modulus of elasticity. This trend highlights the crucial trade-off between producing lightweight concrete and mainta000ining optimal mechanical performance, while also introducing concerns regarding thermal stability due to MC’s high LOI and the presence of graphite and other volatiles. Simultaneously, incorporating MC aggregates enhances sustainability by decreasing reliance on natural resources, saving energy, lowering CO₂ emissions, and diverting industrial by-products from landfills. Although mechanical properties are modestly decreased, these mixes meet requirements for non-structural and lightweight applications, offering a beneficial balance of mechanical adequacy, cost-effectiveness, and environmental advantages in concrete production.

## A comparative study of NWC and SLWC

A very critical factor that helps in choosing the appropriate type of concrete for a certain project is the cost of this concrete. Many factors influence the cost of any type of concrete. Two types of concrete were studied: NWC by using NWA, and SLWC by using MC as a coarse aggregate. A case study and a small application of these types of concrete are presented, and a comparative study was done on the cost of the materials used in both types of concrete mortars and the quantity of steel needed in both case studies, and then the cost of the steel for both types as a structural reinforced concrete to be used in the construction of structural buildings. Moreover, the prices of the materials were collected from suppliers and represent current market conditions. According to the study, the two types of mixtures used in these case studies were 0% MC, which is the NWA, and 75% MC, which is SLWC. The study was done with prices of concrete materials, such as NWA and sand, given in LE per cubic meter, where the price of MC was given in LE per ton.

After the bulk density test of NWA, lightweight aggregate, and sand, their results were 1520 kg/m^3^, 501.33 kg/m^3^, and 1765 kg/m^3^, respectively. Then the prices of the concrete materials were calculated per kg as shown in Table 10 for NWA and sand, and Table 11 for MC. According to the prices of the concrete materials, the total price of the 0% MC mixture was calculated as shown in Table 12 and the total price of the 100% MC mixture was calculated as shown in Table 13.

**Table 10:** Price of NWA and sand.

Concrete materials	Price (LE/m^3^)	Bulk density (kg/m^3^)	Price (LE/kg)
NWA	350	1520	0.23
Sand	200	1765	0.11

**Table 11:** Price of MC.

Concrete materials	Price (LE/ton)	Price (LE/kg)
MC	500	0.5

**Table 12:** Total price of the 0% MC mixture.

Concrete materials	Quantity (kg/m^3^)	Price (LE/kg)	Total price (LE/m^3^)
NWA	1018	0.23	234.14
Sand	738	0.11	81.18
Cement	424	3.00	1272
Water	195	0.1	19.5
Total Price (LE/m^3^)			1606.82

**Table 13:** Total price (LE/m^3^) of the 75% MC mixture.

Concrete materials	Quantity (kg/m^3^)	Price (LE/kg)	Total price (LE/m^3^)
NWA	131.25	0.23	30.19
MC	393.75	0.5	196.9
Sand	592.1	0.11	65.13
Cement	424	3.00	1272
Water	195	0.1	19.5
Total Price (LE/m^3^)			1583.72

From Tables 12 and 13, the total price per cubic meter for NWC is 1606.82 LE/m^3^, while the total price per cubic meter for SLWC is 1583.72 LE/m^3^. This means the SLWC is cheaper than the NWA by 23.1 LE/m^3^.

A practical example of a small structure composed of a one-way solid slab, four beams, two of which were main beams and the other two were secondary, and four columns at the corners of the slab were evaluated to determine the area of reinforced steel. These structural elements were designed as NWC using NWA and were designed as SLWC using MC according to ECP^15^. The NWC with 0% MC has a unit weight equal to 2168 kg/m^3^, while the SLWC with 75% MC has a unit weight equal to 1678 kg/m^3^.

Based on the lower unit weight of SLWC, the total area of reinforced steel was reduced from the normal-weight structure to the lightweight structure. As the weight of concrete of the structural elements decreased, the bending moment acting on them decreased, resulting in a reduction in the area of reinforced steel used for these structural elements. The total reduction of the reinforced steel from the normal-weight structure to the lightweight structure was calculated to be 12% as shown in Table 14.

**Table 14:** The total area of reinforced steel and the total reduction of reinforced steel.

Steel of structural concrete elements	Area steel of NWC (mm^2^)	Area steel of SLWC (mm^2^)
Columns	77.1155	62.74
Main beams bottom	594.70	519.71
Main beams top	194.11	173.24
Secondary beams bottom	33.3	24.95
Secondary beams top	11.1	8.57
Solid slab secondary	81.044	74.39
Solid slab main	405.22	371.95
Total area steel (mm^2^)	1396.5895	1235.55
Total reduction in reinforced steel	12%	

## Conclusions

This study evaluated the mechanical, thermal, and economic feasibility of using metallurgical coal (MC) as a coarse aggregate in structural lightweight concrete (SLWC). The experimental results demonstrate a trade-off between density reduction and mechanical performance, governed by the porous nature of the MC aggregate. Increasing the MC content consistently reduced the unit weight of the concrete from 2168 to 1642 kg/m^3^, successfully meeting the density requirements for structural lightweight concrete. However, this weight reduction was accompanied by a decrease in mechanical properties, with compressive strength dropping from 37.6 to 20.7 MPa and the modulus of elasticity declining significantly as the replacement level increased. Despite the strength loss and degradation observed under elevated temperatures up to 600°C, the MC concrete maintained adequate residual strength to ensure structural stability, validating its thermal viability.

From an economic and sustainable perspective, this research contributes to the field by validating the innovative use of MC—a byproduct distinct from common coal combustion wastes—as a functional lightweight aggregate. The study highlights significant efficiency gains, notably that SLWC with 75% MC is more cost-effective than normal-weight concrete, offering a cost reduction of approximately 23.1 LE/m^3^. Furthermore, the reduced dead load of the MC concrete resulted in a calculated 12% reduction in the required area of steel reinforcement for structural elements. These findings confirm that utilizing MC offers a feasible pathway for sustainable construction by diverting industrial by-products from landfills and reducing transportation energy costs through lighter structural loads.

The practical application of MC concrete is dictated by the replacement percentage, with distinct recommendations for structural and non-structural uses. Mixtures containing 25% to 75% MC satisfy the strength thresholds of ACI 213R-87 for structural lightweight concrete and are recommended for multi-story building elements such as slabs, beams, and composite decking, where dead load reduction is critical. Conversely, the 100% MC mixture, while providing maximum weight reduction, falls below the structural compressive strength threshold of 17.2 MPa and is consequently recommended for non-structural applications, including partition walls, insulation layers, and lightweight blocks.

While the mechanical and thermal viability of MC aggregates has been established, further research is required to fully validate long-term performance and optimize its application. Future studies should focus on conducting scanning electron microscopy (SEM) to investigate the pore structure and the interfacial transition zone (ITZ) to better understand the mechanisms governing strength loss. High-temperature durability tests for the remaining 75% and 100% MC mixtures to complete the thermal performance evaluation across all replacement levels should also be conducted. Additionally, detailed assessments of long-term durability factors, including shrinkage, carbonation, and freeze–thaw resistance, are necessary to ensure the material’s reliability in aggressive environments, while further exploration into using MC-based concrete as a repair material could leverage its lightweight properties to minimize additional loading on deteriorated structures.

## Data Availability

The data are available on request from the corresponding author.
